# Adjuvant chemotherapy for stage III colon cancer: relative dose intensity and survival among veterans

**DOI:** 10.1186/s12885-015-1038-y

**Published:** 2015-02-18

**Authors:** Sherrie L Aspinall, Chester B Good, Xinhua Zhao, Francesca E Cunningham, Bernadette B Heron, Mark Geraci, Vida Passero, Roslyn A Stone, Kenneth J Smith, Renee Rogers, Jenna Shields, Megan Sartore, D Patrick Boyle, Sherry Giberti, John Szymanski, Doug Smith, Allen Ha, Jolynn Sessions, Shawn Depcinski, Shane Fishco, Irvin Molina, Tanja Lepir, Carmela Jean, Lymaris Cruz-Diaz, Jessica Motta, Rebeca Calderon-Vargas, Janelle Maland, Sean Keefe, Marshall Tague, Alice Leone, Brian Glovack, Blair Kaplan, Sean Cosgriff, Lindsay Kaster, Ivy Tonnu-Mihara, Kimmai Nguyen, Jenna Carmichael, Linda Clifford, Kan Lu, Gurkamal Chatta

**Affiliations:** 1 VA Pharmacy Benefits Management Services, Hines, IL USA; 2 Center for Health Equity Research and Promotion, VA Pittsburgh Healthcare System, University Drive (151C), Building 30, Pittsburgh, PA 15240 USA; 3 University of Pittsburgh, School of Pharmacy, Pittsburgh, PA USA; 4 University of Pittsburgh, School of Medicine, Pittsburgh, PA USA; 5 VA Pittsburgh Healthcare System, Pittsburgh, PA USA; 6 University of Pittsburgh Graduate School of Public Health, Pittsburgh, PA USA; 7 Division of Clinical Modeling and Decision Sciences, University of Pittsburgh, Pittsburgh, PA USA; 8 VA Maine Health Care System, Augusta, ME USA; 9 VA Connecticut Healthcare System, West Haven, CT USA; 10 Martinsburg VA Medical Center, Martinsburg, WV USA; 11 Richmond VA Medical Center, Richmond, VA USA; 12 Asheville VA Medical Center, Asheville, NC USA; 13 James A. Haley Veterans Hospital, Tampa, FL USA; 14 Miami VA Medical Center, Miami, FL USA; 15 VA Caribbean Healthcare System, San Juan, PR USA; 16 Harry S. Truman VA Hospital, Columbia, MO USA; 17 Kansas City VA Medical Center, Kansas City, MO USA; 18 Iowa City VA Medical Center, Iowa City, IA USA; 19 Louis Stokes Cleveland VA Medical Center, Cleveland, OH USA; 20 Jesse Brown VA Medical Center, Chicago, IL USA; 21 Portland VA Medical Center, Portland, OR USA; 22 Boise VA Medical Center, Boise, ID USA; 23 Long Beach VA Medical Center, Long Beach, CA USA; 24 VA Sierra Nevada Health Care System, Reno, NV USA; 25 Sacramento VA Medical Center, Mather, CA USA; 26 Virginia Mason Medical Center, Seattle, WA USA

**Keywords:** Colon cancer, Chemotherapy, Relative dose intensity, Survival

## Abstract

**Background:**

Given the paucity of information on dose intensity, the objective of this study is to describe the use of adjuvant chemotherapy for stage III colon cancer, focusing on relative dose intensity (RDI), overall survival (OS) and disease-free survival (DFS).

**Methods:**

Retrospective cohort of 367 patients diagnosed with stage III colon cancer in 2003–2008 and treated at 19 VA medical centers. Kaplan-Meier curves summarize 5-year OS and 3-year DFS by chemotherapy regimen and RDI, and multivariable Cox proportional hazards regression was used to model these associations.

**Results:**

5-fluorouracil/leucovorin (FU/LV) was the most commonly initiated regimen in 2003 (94.4%) and 2004 (62.7%); in 2005–2008, a majority of patients (60%-74%) was started on an oxaliplatin-based regimen. Median RDI was 82.3%. Receipt of >70% RDI was associated with better 5-year OS (p < 0.001) and 3-year DFS (P = 0.009) than was receipt of ≤70% RDI, with 5-year OS rates of 66.3% and 50.5%, respectively and 3-year DFS rates of 66.1% and 52.7%, respectively. In the multivariable analysis of 5-year OS, oxaliplatin + 5-FU/LV (versus 5-FU/LV) (HR = 0.55; 95% CI = 0.34-0.91), >70% RDI at the first year (HR = 0.58; 95% CI = 0.37-0.89) and married status (HR = 0.66; 95% CI = 0.45-0.97) were associated with significantly decreased risk of death, while age ≥75 (versus 55–64) (HR = 2.06; 95% CI = 1.25-3.40), Charlson Comorbidity Index (HR = 1.17; 95% CI = 1.06-1.30), T4 tumor status (versus T1/T2) (HR = 5.88; 95% CI = 2.69-12.9), N2 node status (HR = 1.68; 95% CI = 1.12-2.50) and bowel obstruction (HR = 2.32, 95% CI = 1.36-3.95) were associated with significantly increased risk. Similar associations were observed for DFS.

**Conclusion:**

Patients with stage III colon cancer who received >70% RDI had improved 5-year OS. The association between RDI and survival needs to be examined in studies of adjuvant chemotherapy for colon cancer outside of the VA.

**Electronic supplementary material:**

The online version of this article (doi:10.1186/s12885-015-1038-y) contains supplementary material, which is available to authorized users.

## Background

In patients with stage III colon cancer, oxaliplatin with intravenous 5-fluorouracil and leucovorin (5-FU/LV) or oxaliplatin with capecitabine are the preferred adjuvant chemotherapy regimens [1-4]. However, the survival benefit of adding oxaliplatin may not be as great among the elderly [5], and the use of an oxaliplatin-containing regimen has been shown to decline with age and performance status [5-7].

In addition to the regimen selected, chemotherapy duration and intensity have been associated with survival [8-10]. Specifically, two studies suggested that the duration of fluorouracil adjuvant chemotherapy (i.e., 5–7 months versus 1–4 months and 4–6 cycles versus 1–3 cycles) for stage III colon cancer is associated with improved survival [8,9], and one study of capecitabine adjuvant chemotherapy for colorectal cancer (in which 73% of patients had stage III disease) reported that a relative dose intensity (RDI) of >70% was associated with improved overall survival [10]. However, it remains unclear whether RDI is associated with improved survival among patients receiving adjuvant chemotherapy for stage III colon cancer, especially with the use of oxaliplatin-based regimens.

Having patients complete all intended cycles of chemotherapy without dose reductions can be difficult given the toxicities of the medications and concomitant health problems. Decreased completion of adjuvant chemotherapy has been reported in the elderly (i.e., ≥70 years old) and those with comorbidities [8,11], two characteristics that are common in Veterans. Given the paucity of information on dose intensity, particularly in the context of other factors that can impact survival, our objective is to describe the use of adjuvant chemotherapy for stage III colon cancer in a Veteran population, with a focus on associations between RDI and overall survival (OS) and disease-free survival (DFS). We also assess factors associated with receiving >70% RDI, OS and DFS.

## Methods

### Study setting and population

This is a retrospective cohort study of patients with a diagnosis of stage III colon cancer between 2003 and 2008 at 19 VA medical centers in the U.S.; patients were followed through June 2011. Veterans with colon cancer were identified via a search of local tumor registries or data warehouses; then, pharmacists at each site reviewed VA electronic medical records (i.e., Computerized Patient Record System or CPRS) to ascertain those with pathology confirmed stage III disease [12]. Pharmacists also reviewed CPRS to determine which of these identified patients received adjuvant chemotherapy (defined as receipt within 120 days of surgical resection) in the VA [13]. The Institutional Review Boards for participating sites and VA Pharmacy Benefits Management Services approved the study (see “Competing interests”).

### Data sources and data collection

Using CPRS, pharmacists recorded the date of birth, date of surgical resection, tumor staging (i.e., Tumor, Node and Metastasis), other prognostic characteristics (i.e., preoperative carcinoembryonic antigen [CEA]; histologic type and grade; number of lymph nodes evaluated; number of positive lymph nodes; margins, and presence/absence of lymphovascular invasion, perineural invasion, bowel obstruction and perforation) [14], Eastern Cooperative Oncology Group (ECOG) performance status prior to initiation of chemotherapy, time between surgery and start of chemotherapy, adjuvant chemotherapy regimen administered, adverse drug events (ADEs) that caused a delay or change in chemotherapy, and date of local or distant cancer recurrence. Patient demographic and comorbidity data were obtained from the VA Medical SAS Datasets (Austin Information Technology Center in Austin, TX), and date of death was obtained from the Vital Status file.

### Chemotherapy regimens

The standard adjuvant chemotherapy regimens are listed in Additional file 1: Appendix I. Standard Adjuvant Chemotherapy Regimens for Colon Cancer. We classified the regimens as 5-FU/LV, oxaliplatin plus 5-FU/LV, oxaliplatin plus capecitabine, capecitabine monotherapy and “other” (e.g., regimens containing bevacizumab, irinotecan) based on the active medications.

### RDI

RDI is the proportion of the standard regimen (Additional file 1: Appendix I. Standard Adjuvant Chemotherapy Regimens for Colon Cancer) dose intensity that patients received over their course of chemotherapy. RDI was calculated for each patient according to the method proposed by Hryniuk and Bush [15]. For each drug within each regimen, the total dosage that the patient received was divided by the total dosage specified by the corresponding standard regimen; these proportions were averaged across drugs within a given regimen. If patients switched regimens, the regimen-specific RDIs were summed.

### Primary outcome measures

The primary outcomes were 5-year OS and 3-year DFS. OS was the time from cessation of chemotherapy to death from any cause. DFS was the time from cessation of chemotherapy to either colon cancer recurrence or death, whichever came first; 3-year DFS has been used as a surrogate marker for OS in clinical trials of adjuvant chemotherapy for colon cancer [16,17]. The survival time origin was the date a patient ceased chemotherapy because a key independent variable, RDI, was based on the entire course of adjuvant therapy received.

### Statistical analysis

Patient baseline characteristics, including demographics, comorbidities as defined in the Deyo et al. adaptation of the Charlson Comorbidity Index minus malignancy [18], ECOG performance status, tumor staging and other prognostic factors are described for patients with stage III colon cancer who received adjuvant chemotherapy in the VA, overall and by chemotherapy regimen administered in the first cycle. For the first cycle of chemotherapy, we estimated the proportion of patients who received each regimen by year of pathologically-confirmed diagnosis. Chi-square or Fisher exact tests were used to compare categorical variables across initial regimens, and ANOVA or Kruskal-Wallis tests were used to compare continuous variables. For subsequent analyses of RDI and survival considering the entire course of adjuvant therapy, those patients who switched to a different regimen after the first cycle were classified as receiving “mixed/other” chemotherapy.

In preliminary survival analyses we assessed alternative categorizations of RDI (i.e., <50%, 50%-70%, 71%-84% and 85%+), then collapsed categories that did not differ significantly in terms of their association with either OS or DFS. These analyses confirmed the previously identified RDI cut point of >70%. We compared the proportions of patients who received ≤70% vs. >70% RDI by regimen, using Chi-square tests. Multivariable logistic regression was used to assess factors associated with the receipt of >70% RDI. We summarized ADE rates overall and by chemotherapy regimen.

Kaplan-Meier survival curves summarize 5-year OS and 3-year DFS by chemotherapy regimen and RDI. Log-rank tests were used to compare subgroups. Multivariable Cox proportional hazards regression was used to evaluate associations between independent variables of interest and survival outcomes. Independent variables suggestive of a bivariate association (i.e., P < 0.15) were included in the initial multivariable models; the final models included only variables with P < 0.05 for either OS or DFS. Chemotherapy regimen, RDI, age, sex, ethnicity/race, Charlson Comorbidity Index, days between surgery and start of chemotherapy and fixed effects for site were forced into both models. We tested the proportional-hazards assumption using time-dependent covariates. Because this assumption was violated for RDI >70% in the model for 5-year OS, an interaction term between RDI >70% and log (year) was added, and annual time-dependent hazard ratios were estimated using linear combinations of model parameters. We also tested interactions between regimens and RDI >70% in both models. All p-values are two-sided. Data management was done using SAS software (Cary, NC) version 9.2. Fisher Exact tests were done using Monte Carlo in Cytel Studio 7 (Cambridge, MA), and all models were run in Stata (College Station, TX) version 11.

## Results and discussion

### Study population

Between 2003 and 2008, 581 patients with pathologically confirmed stage III colon cancer were treated at the 19 participating VA medical centers. Of these patients, 367 (63.2%) received chemotherapy in the VA within 120 days of surgical resection. The most common reasons that patients did not receive chemotherapy in the VA were patient refused (32%), comorbidities (23%), poor performance status (18%) and chemotherapy prescribed by non-VA physician (14%). Few baseline characteristics varied by initial adjuvant chemotherapy regimen (Table 1). Those who initiated capecitabine monotherapy tended to be older and relatively more likely to have an ECOG performance status of 2 to 4.

**Table 1:** **Baseline characteristics of patients with stage III colon cancer who received adjuvant chemotherapy in VA, categorized by regimen administered in the first cycle**

Characteristic	VA Chemotherapy	5-FU/LV	Oxaliplatin plus 5-FU/LV	Oxaliplatin plus Capecitabine	Capecitabine Monotherapy	Other	p-value^a^
	N = 367, N (col%)	N = 126, N (col%)	N = 152, N (col%)	N = 30, N (col%)	N = 48, N (col%)	N = 11, N (col%)	
**Age (mean, SD)**	66.4 (9.9)	67.2 (9.3)	63.7 (9.6)	65.6 (9.2)	73.1 (8.5)	66.3 (12.8)	<0.001
**Age**							<0.001
<55	39 (10.6)	11 (8.7)	23 (15.1)	3 (10.0)	0 (0.0)	2 (18.2)	
55-64	131 (35.7)	41 (32.5)	66 (43.4)	13 (43.3)	8 (16.7)	3 (27.3)	
65-74	110 (30.0)	45 (35.7)	38 (25.0)	7 (23.3)	17 (35.4)	3 (27.3)	
75+	87 (23.7)	29 (23.0)	25 (16.4)	7 (23.3)	23 (47.9)	3 (27.3)	
**Male**	360 (98.1)	124 (98.4)	148 (97.4)	30 (100.0)	47 (97.9)	11 (100.0)	0.86
**Married**							0.92
No	181 (49.3)	62 (49.2)	75 (49.3)	16 (53.3)	24 (50.0)	4 (36.4)	
Yes	184 (50.1)	64 (50.8)	75 (49.3)	14 (46.7)	24 (50.0)	7 (63.6)	
Missing	2 (0.5)	0 (0.0)	2 (1.3)	0 (0.0)	0 (0.0)	0 (0.0)	
**Race/Ethnicity**							<0.001
Hispanic	58 (15.8)	18 (14.3)	32 (21.1)	1 (3.3)	2 (4.2)	5 (45.5)	
Black (non-Hispanic)	59 (16.1)	20 (15.9)	25 (16.4)	3 (10.0)	7 (14.6)	4 (36.4)	
White (non-Hispanic)	237 (64.6)	83 (65.9)	90 (59.2)	23 (76.7)	39 (81.3)	2 (18.2)	
Other/missing	13 (3.5)	5 (4.0)	5 (3.3)	3 (10.0)	0 (0.0)	0 (0.0)	
**Charlson Comorbidity Index** ^**b**^ **(mean, SD)**	1.1 (1.7)	1.3 (1.8)	1.1 (1.9)	0.8 (1.4)	1.2 (1.2)	1.1 (1.0)	0.14
**Tumor**							0.52
T1	16 (4.4)	4 (3.2)	7 (4.6)	1 (3.3)	3 (6.3)	1 (9.1)	
T2	45 (12.3)	15 (11.9)	16 (10.5)	5 (16.7)	9 (18.8)	0 (0.0)	
T3	275 (74.9)	94 (74.6)	119 (78.3)	23 (76.7)	31 (64.6)	8 (72.7)	
T4	31 (8.4)	13 (10.3)	10 (6.6)	1 (3.3)	5 (10.4)	2 (18.2)	
**Node**							0.51
N1	235 (64.0)	87 (69.0)	96 (63.2)	18 (60.0)	28 (58.3)	6 (54.5)	
N2	132 (36.0)	39 (31.0)	56 (36.8)	12 (40.0)	20 (41.7)	5 (45.5)	
**Histologic type**							<0.001
Adenocarcinoma	340 (92.6)	118 (93.7)	147 (96.7)	21 (70.0)	45 (93.8)	9 (81.8)	
Unknown	24 (6.5)	8 (6.3)	3 (2.0)	9 (30.0)	3 (6.3)	1 (9.1)	
Other	3 (0.8)	0 (0.0)	2 (1.3)	0 (0.0)	0 (0.0)	1 (9.1)	
**Histologic grade**							0.68
Well differentiated	35 (9.5)	15 (11.9)	10 (6.6)	2 (6.7)	7 (14.6)	1 (9.1)	
Moderately differentiated	247 (67.3)	81 (64.3)	107 (70.4)	23 (76.7)	29 (60.4)	7 (63.6)	
Poorly differentiated	68 (18.5)	25 (19.8)	27 (17.8)	4 (13.3)	10 (20.8)	2 (18.2)	
Undifferentiated	4 (1.1)	0 (0.0)	4 (2.6)	0 (0.0)	0 (0.0)	0 (0.0)	
Unknown	13 (3.5)	5 (4.0)	4 (2.6)	1 (3.3)	2 (4.2)	1 (9.1)	
**Number of lymph nodes evaluated**							0.03
<12	132 (36.0)	53 (42.1)	48 (31.6)	8 (26.7)	19 (39.6)	4 (36.4)	
12+	229 (62.4)	68 (54.0)	104 (68.4)	22 (73.3)	29 (60.4)	6 (54.5)	
Missing	6 (1.6)	5 (4.0)	0 (0.0)	0 (0.0)	0 (0.0)	1 (9.1)	
**Number of lymph nodes evaluated (mean, SD)**	15.6 (8.9)	14.3 (8.5)	16.6 (9.2)	16.5 (10.0)	15.1 (8.8)	15.3 (7.0)	0.11
**Number of positive lymph nodes (mean, SD) (N = 366)**	3.8 (4.0)	3.4 (3.8)	3.9 (3.9)	4.8 (5.2)	3.6 (3.5)	6.0 (5.7)	0.10
**Preoperative CEA**							0.68
<5 ng/ml	178 (48.5)	60 (47.6)	78 (51.3)	14 (46.7)	20 (41.7)	6 (54.5)	
≥5 ng/ml	84 (22.9)	32 (25.4)	36 (23.7)	6 (20.0)	8 (16.7)	2 (18.2)	
Missing	105 (28.6)	34 (27.0)	38 (25.0)	10 (33.3)	20 (41.7)	3 (27.3)	
**Preoperative CEA (mean, SD) (N = 262)**	8.0 (15.2)	9.4 (18.8)	7.8 (14.2)	4.8 (6.3)	7.1 (11.5)	5.2 (6.3)	0.48
**Lymphovascular invasion**							0.003
Yes	134 (36.5)	41 (32.5)	62 (40.8)	10 (33.3)	17 (35.4)	4 (36.4)	
No	103 (28.1)	26 (20.6)	41 (27.0)	17 (56.7)	17 (35.4)	2 (18.2)	
Unknown	130 (35.4)	59 (46.8)	49 (32.2)	3 (10.0)	14 (29.2)	5 (45.5)	
**Perineural invasion**							0.01
Yes	38 (10.4)	8 (6.3)	17 (11.2)	4 (13.3)	6 (12.5)	3 (27.3)	
No	102 (27.8)	28 (22.2)	45 (29.6)	15 (50.0)	13 (27.1)	1 (9.1)	
Unknown	227 (61.9)	90 (71.4)	90 (59.2)	11 (36.7)	29 (60.4)	7 (63.6)	
**Bowel obstruction**							0.89
Yes	75 (20.4)	27 (21.4)	26 (17.1)	7 (23.3)	13 (27.1)	2 (18.2)	
No	168 (45.8)	56 (44.4)	73 (48.0)	14 (46.7)	21 (43.8)	4 (36.4)	
Unknown	124 (33.8)	43 (34.1)	53 (34.9)	9 (30.0)	14 (29.2)	5 (45.5)	
**Perforation**							0.19
Yes	21 (5.7)	13 (10.3)	5 (3.3)	2 (6.7)	0 (0.0)	1 (9.1)	
No	229 (62.4)	74 (58.7)	94 (61.8)	20 (66.7)	34 (70.8)	7 (63.6)	
Unknown	117 (31.9)	39 (31.0)	53 (34.9)	8 (26.7)	14 (29.2)	3 (27.3)	
**Margins**							0.003
All margins histologically negative	307 (83.7)	100 (79.4)	138 (90.8)	20 (66.7)	39 (81.3)	10 (90.9)	
1 or more margins included	25 (6.8)	9 (7.1)	10 (6.6)	2 (6.7)	4 (8.3)	0 (0.0)	
Unknown	35 (9.5)	17 (13.5)	4 (2.6)	8 (26.7)	5 (10.4)	1 (9.1)	
**ECOG performance status** ^**c**^							0.006
0	96 (26.2)	26 (20.6)	52 (34.2)	7 (23.3)	6 (12.5)	5 (45.5)	
1	44 (12.0)	19 (15.1)	14 (9.2)	2 (6.7)	8 (16.7)	1 (9.1)	
2 -4	20 (5.4)	8 (6.3)	3 (2.0)	1 (3.3)	7 (14.6)	1 (9.1)	
Missing or unknown	207 (56.4)	73 (57.9)	83 (54.6)	20 (66.7)	27 (56.3)	4 (36.4)	
**Days between surgery and start of chemotherapy**							0.053
≤30	12 (3.3)	5 (4.0)	4 (2.6)	0 (0.0)	3 (6.3)	0 (0.0)	
31-60	232 (63.2)	95 (75.4)	85 (55.9)	19 (63.3)	27 (56.3)	6 (54.5)	
61-90	90 (24.5)	21 (16.7)	45 (29.6)	7 (23.3)	13 (27.1)	4 (36.4)	
91-120	33 (9.0)	5 (4.0)	18 (11.8)	4 (13.3)	5 (10.4)	1 (9.1)	

5-FU/LV = 5-fluouracil/leucovorin; CEA = carcinoembryonic antigen; ECOG = Eastern Cooperative Oncology Group.

^a^Chi-square tests or Fisher exact tests for categorical variables and ANOVA for continuous variable of age.

^b^Malignancy was removed from the Charlson Comorbidity Index because all patients have colon cancer.

^c^ECOG performance status prior to initiation of chemotherapy.

### Adjuvant chemotherapy

**Figure 1:**
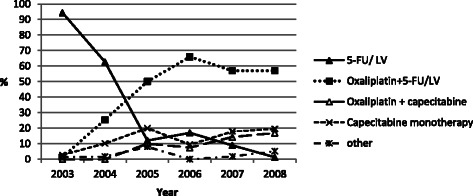
**Adjuvant chemotherapy regimen received in the*first cycle*by year of pathology confirmed diagnosis.** “Other” includes 3 patients who received an oxaliplatin-based regimen plus bevacizumab; 6 patients who received an irinotecan-based regimen, and 2 patients who received a regimen not listed.

The most commonly initiated regimen was 5-FU/LV in 2003 (94.4%) and 2004 (62.7%), while a majority of patients (60%-74%) started an oxaliplatin-based regimen in 2005–2008 (50%-66% received oxaliplatin plus 5-FU/LV) (Figure 1). At some point after their first cycle, 57 (15.5%) patients were switched to different adjuvant chemotherapy regimens, including 26 who started oxaliplatin plus 5-FU/LV. Considering the entire course of adjuvant therapy, 30.8% received 5-FU/LV; 34.3% received oxaliplatin plus 5-FU/LV; 5.4% received oxaliplatin plus capecitabine; 12.5% received capecitabine monotherapy, and 16.9% received “mixed” (i.e., they switched between regimens) or “other” regimens.

### Relative dose intensity

Based on standard adjuvant chemotherapy regimens (Additional file 1: Appendix I. Standard Adjuvant Chemotherapy Regimens for Colon Cancer), the median RDI was 82.3%, and 56.1% of patients completed >70% RDI (Table 2). Overall, 54.9% of patients completed all chemotherapy cycles, regardless of dose, and median time on chemotherapy was 5.4 months. The percentage of patients completing >70% RDI ranged from 71.4% for those who received oxaliplatin plus 5-FU/LV to 40% and 30.4%, respectively, for those who received oxaliplatin plus capecitabine or capecitabine monotherapy. In the multivariable model, the odds of receiving >70% RDI were significantly lower in patients below age 55 and above age 64 (versus 55–64 years of age) (age < 55: OR 0.34; 95% CI 0.14, 0.85; age 65–74: OR 0.46; 95% CI 0.24, 0.90; age ≥75: OR 0.31; 95% CI 0.15, 0.65) and among those who received capecitabine monotherapy (OR 0.27; 95% CI 0.12, 0.61 relative to 5-FU/LV). No other factors considered were significantly associated with completing >70% RDI (Additional file 1: Appendix II. Multivariable Model of Factors Associated with Receiving >70% RDI).

**Table 2:** **Relative Dose Intensity (RDI), patients completing all cycles of chemotherapy and months of chemotherapy by regimen**

Outcomes	Total	5-FU/LV	Oxaliplatin plus 5-FU/LV	Oxaliplatin plus Capecitabine	Capecitabine Monotherapy	Mixed/Other^a^
	N = 367	N = 113	N = 126	N = 20	N = 46	N = 62
**Patients completing ≤70% of RDI, n (col%)**	122 (33.2)	35 (31.0)	25 (19.8)	11 (55.0)	30 (65.2)	21 (33.9)
**Patients completing >70% of RDI, n (col%)**	206 (56.1)	70 (61.9)	90 (71.4)	8 (40.0)	14 (30.4)	24 (38.7)
**Missing or unknown,** ^**b**^ **n (col%)**	39 (10.6)	8 (7.1)	11 (8.7)	1 (5.0)	2 (4.3)	17 (27.4)
**RDI (%), median (IQR)**	82.3 (49.7, 97.5)	96.7 (52.0, 100)	86.1 (70.9, 96.3)	62.2 (46.9, 81.4)	51.2 (29.2, 72.1)	68.8 (42.8, 90.2)
**Patients completing all cycles (N = 348), n (row%)**	191 (54.9)	79 (73.8)	71 (58.2)	6 (30.0)	18 (40.0)	17 (31.5)
**Months of chemotherapy, median (IQR)**	5.4 (4.3, 6.2)	5.2 (4.5, 6.6)	5.6 (4.9, 6.1)	4.5 (3.1, 5.6)	4.9 (2.1, 5.6)	5.5 (3.6, 6.4)

IQR = interquartile range (25th percentile, 75th percentile).

^a^“Mixed/other” includes patients who switched regimens and those who received a chemotherapy regimen not listed.

^b^39 (10.6%) categorized as missing because the regimen was not standard, or regimen data were missing. Therefore, RDI could not be calculated.

Note: P < 0.0001 for the difference across regimens in categorical variables of RDI and patients completing all cycles of chemotherapy using Fisher exact tests and Chi-square tests, respectively; P < 0.001 for differences across regimens in continuous RDI, and P = 0.007 for differences in length of chemotherapy using Kruskal-Wallis tests.

### Adverse drug events

Among the 367 patients, 259 (70.6%) reported a total of 660 ADEs that caused a delay or change in chemotherapy. The most common ADEs included neutropenia (N = 154, 23.3% of total ADEs), diarrhea/gastrointestinal toxicity (N = 134, 21.3%) and thrombocytopenia (N = 114, 17.3%) (Additional file 1: Appendix III. Number of Adverse Drug Events and Rate per 10 Cycles by Regimen). At the episode level, overall ADE rates were similar across regimens (2.2-2.5 per 10 cycles of chemotherapy), although some individual ADE rates (e.g., neutropenia, hand-foot syndrome) differed across regimens. ADEs were reported by relatively more patients who received oxaliplatin plus 5-FU/LV (78.7%) and relatively fewer patients who received 5-FU/LV (49.4%); corresponding figures ranged from 65%-69% for the other regimens (data not tabled).

### Overall survival and disease-free survival

**Figure 2:**
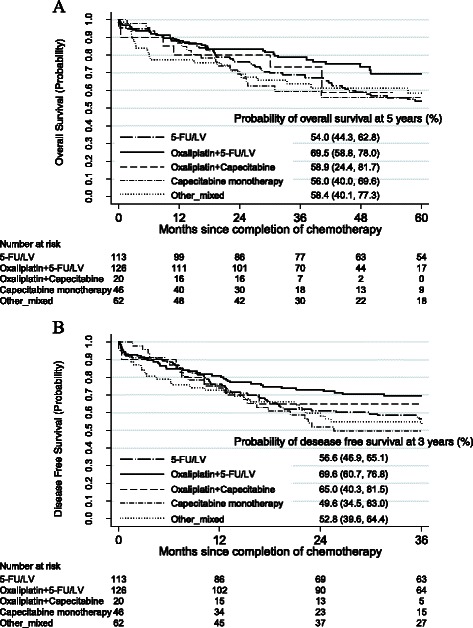
**Kaplan-Meier estimates of survival by chemotherapy regimen received. A**. Kaplan-Meier estimates of overall survival by chemotherapy regimen received. **B**. Kaplan-Meier estimates of disease-free survival by chemotherapy regimen received.

**Figure 3:**
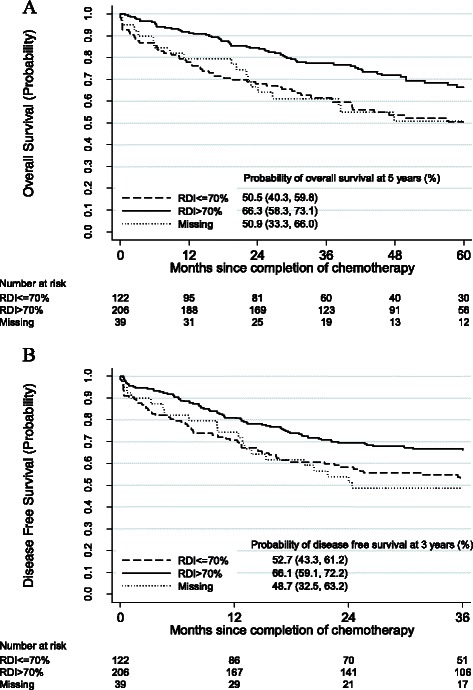
**Kaplan-Meier estimates of survival by relative dose intensity. A**. Kaplan-Meier estimates of overall survival by relative dose intensity. **B**. Kaplan-Meier estimates of disease-free survival by relative dose intensity.

Of the 367 patients who received adjuvant chemotherapy in the VA during the study period, 132 (36.0%) died by year 5, and 146 (39.8%) died or had a recurrence of their colon cancer by year 3. Oxaliplatin plus 5-FU/LV was associated with better OS than was 5-FU/LV (p = 0.04); the 5-year OS rates were 69.5% and 54.0%, respectively (Figure 2A). Similar estimates were obtained for 3-year DFS (69.6% and 56.6%, respectively), and between-regimen differences in DFS were of borderline statistical significance (P = 0.06, Figure 2B). Receipt of >70% RDI was associated with better 5-year OS (p < 0.001) and 3-year DFS (P = 0.009) than was receipt of ≤70% RDI, with 5-year OS of 66.3% and 50.5%, respectively and 3-year DFS rates of 66.1% and 52.7%, respectively (Figures 3A and B). The Kaplan-Meier plot of OS also indicates some attenuation over time in the apparently protective effect of >70% RDI.

In the multivariable analysis of factors associated with 5-year OS (Table 3), oxaliplatin plus 5-FU/LV (versus 5-FU/LV) (HR 0.55; 95% CI 0.34, 0.91), >70% RDI at the *first* year (HR 0.58; 95% CI 0.37, 0.89) and being married (HR 0.66; 95% CI 0.45, 0.97) were associated with decreased mortality, while age ≥75 (versus 55–64 years of age) (HR 2.06; 95% CI 1.25, 3.40), Charlson Comorbidity Index (HR 1.17; 95% CI 1.06, 1.30), T4 tumor status (versus T1/T2) (HR 5.88; 95% CI 2.69, 12.9), N2 node status (HR 1.68; 95% CI 1.12, 2.50) and bowel obstruction (HR 2.32, 95% CI 1.36, 3.95) were associated with increased mortality. In the multivariable model of 3-year DFS, similar associations were observed between most of these factors and cancer recurrence/death prior to recurrence; no significant differences were identified by regimen. Interactions between regimen and receipt of >70% RDI were not statistically significant in either model (P > 0.20 for each).

**Table 3:** **Multivariable cox models for overall and disease-free survival** ^a^

	Overall survival (5 years)^b^		Disease free survival^c^(3 years)	
	HR (95% CI)	P values	HR (95% CI)	P values
**Chemotherapy regimens**				
5-FU/ LV	Reference		Reference	
Oxaliplatin plus 5-FU/LV	0.55 (0.34,0.91)	0.02	0.65 (0.41,1.05)	0.08
Oxaliplatin plus Capecitabine	1.15 (0.42,3.16)	0.79	1.07 (0.42,2.70)	0.89
Capecitabine Monotherapy	0.80 (0.43,1.50)	0.50	0.91 (0.50,1.64)	0.75
Mixed/Other	0.89 (0.50,1.59)	0.70	1.20 (0.70,2.05)	0.51
**Relative dose intensity**				
≤70%	Reference		Reference	
>70%	NA^d^		0.75 (0.50,1.11)	0.15
At year 1	0.58 (0.37,0.89)	0.01		
At year 2	0.74 (0.47,1.18)	0.21		
At year 3	0.86 (0.52,1.44)	0.57		
At year 4	0.96 (0.54,1.68)	0.88		
At year 5	1.04 (0.56,1.91)	0.90		
Unknown or Missing	0.95 (0.51,1.78)	0.88	0.97 (0.53,1.77)	0.91
**Age (years)**				
<55	0.68 (0.30, 1.52)	0.34	1.00 (0.50, 1.97)	0.99
55-64	Reference		Reference	
65-74	0.99 (0.61,1.62)	0.96	1.14 (0.72,1.82)	0.57
75+	2.06 (1.25,3.40)	0.005	1.98 1.21,3.23)	0.006
**Male**	1.70 (0.39,7.52)	0.48	1.67 (0.39,7.27)	0.49
**Race/Ethnicity**				
White (non-Hispanic)	Reference		Reference	
Hispanic	0.56 (0.17,1.80)	0.33	0.27 (0.08,0.93)	0.04
Black (non-Hispanic)	0.75 (0.37,1.50)	0.41	0.76 (0.40,1.44)	0.40
Other/Missing	1.42 (0.58,3.48)	0.44	1.35 (0.59,3.11)	0.48
**Charlson Comorbidity Index (per unit)**	1.17 (1.06,1.30)	0.002	1.15 (1.04,1.27)	0.007
**Married**	0.66 (0.45,0.97)	0.03	0.86 (0.59,1.24)	0.42
**Tumor**				
T1/T2^e^	Reference		Reference	
T3	1.62 (0.83,3.14)	0.15	1.63 (0.88,3.02)	0.12
T4	5.88 (2.69,12.9)	<0.001	5.84 (2.82,12.1)	<0.001
**Node**				
N1	Reference		Reference	
N2	1.68 (1.12,2.50)	0.01	1.89 (1.30,2.77)	0.001
**Lymphovascular invasion**				
No	Reference		Reference	
Yes	1.69 (0.90,3.17)	0.10	1.95 (1.10,3.45)	0.02
Unknown or Missing	1.09 (0.57,2.07)	0.79	1.20 (0.66,2.16)	0.55
**Bowel obstruction**				
No	Reference		Reference	
Yes	2.32 (1.36,3.95)	0.002	2.12 (1.28,3.51)	0.003
Unknown or Missing	1.05 (0.57,1.95)	0.88	1.19 (0.67,2.10)	0.56
**Days between surgery and start of chemotherapy**				
≤30	Reference		Reference	
31-60	1.44 (0.40,5.22)	0.58	1.14 (0.37,3.49)	0.82
61-90	1.65 (0.43,6.25)	0.47	1.35 (0.42,4.33)	0.61
91-120	1.84 (0.46,7.41)	0.39	1.56 (0.46,5.32)	0.47

^a^Variable selection: Predictor variables that were suggestive of a bivariate association (i.e., P < 0.15) were included in the initial multivariable model, and then the final models included only variables with P < 0.05 in either model. Chemotherapy regimens, RDI, age, sex, ethnicity/race, Charlson Comorbidity Index, days between surgery and start of chemotherapy, and fixed effects for site were forced into both models.

^b^A hazard ratio (HR) >1 indicates the covariate is associated with an increased risk of death from any cause, and therefore, decreased overall survival.

^c^A HR > 1 indicates the covariate is associated with an increased risk of colon cancer recurrence or death, and therefore, decreased disease-free survival.

^d^The proportional-hazards assumption does not hold for RDI in the overall survival model. The log hazard of RDI > 70% at time 0 is −0.52 (95% CI −0.94, −0.09), and the log hazard for the interaction between time (year) and RDI > 70% is 0.37 (95% CI 0.07, 0.66), P = 0.01, which indicates that the protective effect of RDI > 70% attenuates over time.

^e^T1 andT2 were collapsed because of small sample size, and both are typically together in the anatomic staging/prognostic groups of stage III colon cancer.

## Discussion

Our study fills an important void in the literature regarding an association between RDI and 5-year OS among patients receiving adjuvant chemotherapy for stage III colon cancer. In addition, our study comprehensively evaluated other factors that have been associated with survival, including demographics, comorbidities, tumor pathology, clinical findings, preoperative CEA and chemotherapy regimen due to the richness of the VA electronic medical record. [2,3,14,19] Veterans predominantly received adjuvant chemotherapy regimens that were recommended at the time for stage III colon cancer; once the initial results of the MOSAIC trial were published, oxaliplatin + 5-FU/LV was prescribed for the majority of patients [2]. That very few patients received bevacizumab mirrors evidence-based practice [20,21]. VA adjuvant chemotherapy use is consistent with that reported outside of the VA [7]. Our “real-world” observation that older and “sicker” (i.e., higher ECOG performance status) patients were more likely to receive capecitabine monotherapy, and less likely to receive oxaliplatin + 5FU/LV, is consistent with other studies that have reported decreased use of oxaliplatin-based regimens among the elderly and those with poor performance status [5-7]. Physicians may have been concerned about the ability of these patients to tolerate the more serious toxicities of oxaliplatin.

Increased age and comorbidity also can contribute to decreased completion of chemotherapy [8,11,19], and a shorter duration of chemotherapy has been associated with poorer survival in stage III colon cancer. Other studies have reported that patients who received 5–7 months of 5-FU/LV had lower overall mortality than those who received 1–4 months (HR 0.59; 95% CI 0.49, 0.71) [8], and that patients who failed to complete 4–6 cycles of 5-FU/LV had higher cancer-specific mortality (HR 2.24; 95% CI 1.66, 3.03) [9]. However, these studies did not consider the chemotherapy dose. One study published in abstract form examined the association between capecitabine dose intensity and survival in colorectal cancer patients and reported that patients receiving >70% RDI had improved relapse-free survival (HR 0.37; 95% CI 0.17-0.82) and OS (HR 0.35; 95% CI 0.14-0.88) [10]. An RDI of >70% also has been associated with improved 5-year survival in non-Hodgkin’s lymphoma [22,23]. Similarly, we found that >70% RDI was associated with both 3-year DFS and 5-year OS in unadjusted Cox proportional hazards models and 5-year OS in the multivariable analysis. The benefit was seen only in the first year after the completion of chemotherapy. This attenuation is likely related to the influence of comorbidities on survival among a more elderly population receiving chemotherapy in the adjuvant setting. Our median RDIs for 5-FU/LV and oxaliplatin plus 5-FU/LV are similar to those reported in the randomized controlled trial of 5-FU/LV alone or oxaliplatin plus 5-FU/LV as adjuvant treatment for colon cancer (MOSAIC trial) (i.e., 97.7% for 5-FU alone; 80.5% for oxaliplatin and 84.4% for 5-FU in the group given oxaliplatin plus 5-FU/LV [2].

Although previously published studies have reported an association between age, marital status and comorbidities and completion of chemotherapy [8,11], we did not observe similar associations with receipt of RDI >70%. Perhaps, physicians considered some of these factors when discussing chemotherapy options with patients. There was a 73% decrease in the odds of receiving >70% RDI in those who took capecitabine monotherapy, and the point estimate was comparable in those who received oxaliplatin plus capecitabine. Our dosing data were obtained primarily from pharmacy dispensing records and do not account for doses that were not taken unless that was documented in the oncology notes; we may be overestimating the actual proportion of patients completing >70% RDI. Noncompliance with capecitabine has been reported and illustrates the need to ask patients about adherence [24,25].

Our 3-year DFS rate for 5-FU/LV is slightly lower than that reported in the MOSAIC trial for patients with stage III disease, but similar for oxaliplatin plus 5-FU/LV (65.3% for 5-FU/LV and 72.2% for oxaliplatin plus 5-FU/LV) [2]. Likewise, our 5-year OS rate for 5-FU/LV is slightly lower than the 6-year OS rate in MOSAIC, but similar for oxaliplatin plus 5-FU/LV (68.7% for 5-FU/LV and 72.9% for oxaliplatin plus 5-FU/LV) [3]. This is likely related to patient population differences (e.g., age and comorbidities), especially those who received 5-FU/LV alone, and to some extent, our choice of time origin.

Similar factors were associated with 5-year OS and 3-year DFS in our multivariable models. Although some variables did not reach statistical significance in both models, the point estimates were comparable. Consistent with MOSAIC trial results, improved OS was seen in patients who received oxaliplatin + 5-FU/LV as compared with 5-FU/LV alone [3]. This is important because of the factors associated with OS, only regimen and RDI are potentially modifiable. Even after adjusting for these two variables and other prognostic factors, age ≥ 75 years old, having more comorbidities and not being married were associated with decreased OS. Although survival benefit with adjuvant chemotherapy in elderly patients with stage III colon cancer has been reported [5], such patients may have more coexisting conditions that limit OS compared with younger patients [9,26]. The number of comorbidities has been associated with decreased survival in other studies of colon cancer [27,28]. Finally, being married may contribute to improved overall survival because of better emotional support. In a recent analysis of marital status and cancer-related mortality, which included colorectal cancer, unmarried patients had a higher risk of death from their cancer [29].

Although our study was comprehensive in its assessment of factors associated with survival, there are potential limitations. First, we did not collect data on subsequent chemotherapy among those who had cancer recurrences. This could have positively or negatively affected 5-year OS depending upon the proportion who were treated for the recurrence. Second, while receipt of >70% RDI was not significantly associated with 3-year DFS in the multivariable model, the point estimate did suggest a potential protective effect; in addition, we did observe a significant unadjusted association. The statistical non-significance of the adjusted association could be due to a small sample size. Third, patients who received capecitabine monotherapy were older on average and had worse performance status than those who received other adjuvant regimens. This may have contributed to relatively fewer of them receiving >70% RDI; however, DFS and OS with capecitabine monotherapy were not significantly different from 5-FU/LV in our study. Fourth, our population was predominantly male, so our results may not apply to females. However, sex was not independently associated with survival in a study of adjuvant chemotherapy in the community [26]. Finally, our study was observational, and outcomes may have been influenced by unmeasured clinical characteristics.

## Conclusions

In conclusion, we found that patients with stage III colon cancer who received >70% RDI had improved 5-year OS after adjusting for other prognostic factors. We believe our study offers powerful “real world” data that demonstrate not only the effectiveness of adjuvant chemotherapy for stage III colon cancer, but also underscore the importance of administering recommended doses. The association between RDI and survival needs to be examined in studies of adjuvant chemotherapy for colon cancer outside of the VA.

## Additional file

Additional file 1: **Appendix I.** Standard Adjuvant Chemotherapy Regimens for Colon Cancer. **Appendix II.** Multivariable Model of Factors Associated with Receiving >70% RDI. **Appendix III.** Number of Adverse Drug Events and Rate per 10 Cycles by Regimen.
