# Supplementary material for: Adjuvant chemotherapy for stage III colon cancer: relative dose intensity and survival among veterans
Source: BMC Cancer. 2015 Feb 18;15:62. doi: 10.1186/s12885-015-1038-y (PMC4352567; doi:10.1186/s12885-015-1038-y)
Supplement: Additional file 1: — Appendix I. Standard Adjuvant Chemotherapy Regimens for Colon Cancer. Appendix II. Multivariable Model of Factors Associated with Receiving >70% RDI. Appendix III. Number of Adverse Drug Events and Rate per 10 Cycles by Regimen. [file 12885_2015_1038_MOESM1_ESM.docx]

Appendix I. Standard Adjuvant Chemotherapy Regimens for Colon Cancer

**5-FU + LV Regimens**

**5-FU + LV (Roswell park regimen)**
5-FU 500 mg/m2 iv bolus 1 h after the start of leucovorin
Leucovorin 500 mg/m2 iv over 2 hrs
Qw x 6 wks every 8 wks for 3-4 cycles

**5-FU + LV (Mayo clinic regimen)**
5-FU 370-425 mg/m2/d iv bolus d1-5
Leucovorin 20-25 mg/m2/d iv bolus d1-5
Q4w x 6 cycles

**Modified deGramont**

**Leucovorin 400 mg/m2 IV over 2 hours on day 1;**

**5-FU 400 mg/m2 IV bolus x 1 on day 1; followed by**

**5-FU 2400 mg/m2 IV over 46 hours; repeat every 2 weeks x 12 cycles**

**12 cycles**

**Oxaliplatin + 5-FU + LV Regimens**

**FOLFOX4**
Leucovorin 200 mg/m2 iv over 2 hrs before 5-FU, d1 and 2
5-FU 400 mg/m2 iv bolus and then 600 mg/m2 iv over 22 hrs, d1 and d2
Oxaliplatin (Eloxatin) 85 mg/m2 iv d1
Q2w x 12 cycles

**FOLFOX6**
Leucovorin 400 mg/m2 iv over 2 hrs before 5-FU d1
5-FU 400 mg/m2 iv bolus d1 followed by 2400 mg/m2 iv over 46 hrs
Oxaliplatin (Eloxatin) 100 mg/m2 in 500 ml dextrose 5% iv over 2 hours d1
Q2w x 12 cycles

**Modified FOLFOX6**
Leucovorin 400 mg/m2 iv over 2 hrs before 5-FU d1
5-FU 400 mg/m2 iv bolus d1 followed by 2400 mg/m2 iv over 46 hrs
Oxaliplatin (Eloxatin) 85 mg/m2 iv d1
Q2w x 12 cycles

**FLOX**
5-FU 500 mg/m2 iv bolus 1 hr after start of leucovorin qw x 6 weeks every 8 weeks for 3 cycles
Leucovorin 500 mg/m2 iv over 2 hrs qw x 6 weeks every 8 weeks for 3 cycles
Oxaliplatin (Eloxatin) 85 mg/m2 iv over 2 hrs before 5-FU and Leucovorin week 1, 3, 5 of each 8-week cycle for 3 cycles

**Oxalipatin +Capecitabine Regimen**

**XELOX**
Capecitabine (Xeloda) 1000 mg/m2 po bid x 14 days
Oxaliplatin (Eloxatin) 130 mg/m2 iv over 2 hrs d1
Q3w x 8 cycles

**Capecitabine Monotherapy Regimen**

**Capecitabine**
Capecitabine (Xeloda) 1250 mg/m2 po bid x 14 days*
Q3w x 8 cycles

*For patients with renal disease (identified by ICD-9 code) on capecitabine monotherapy, we reduced the standard dosage from 1250mg/m2 to 1000mg/m2 (N=4 patients).

**Other Regimens**

**IFL**

**Irinotecan 125 mg/m2 IV over 90 minutes;**

**Leucovorin 20 mg/m2 IV bolus;**

**5-FU 500 mg/m2 IV bolus; repeat weekly x 4 of 6 week cycles**

**5 cycles total (30 weeks)**

**FOLFIRI**

**Irinotecan 180 mg/m2 IV on day 1;**

**Leucovorin 400 mg/m2 IV over 2 hours on day 1;**

**5-FU 400mg/m2 IV bolus x 1 on day 1; followed by**

**5-FU 2400-3000 mg/m2 over 46 hours; repeat every 2 weeks**

**12 cycles total (6 months)**

**Bev + FOLFOX4**

**Bevacizumab 5 mg/kg IV every 2 weeks;**
Leucovorin 200 mg/m2 iv over 2 hrs before 5-FU, d1 and 2
5-FU 400 mg/m2 iv bolus and then 600 mg/m2 iv over 22 hrs, d 1 and d2  
Oxaliplatin (Eloxatin) 85 mg/m2 iv d1
Q2w x 12 cycles

**Bev + Modified FOLFOX6**

**Bevacizumab 5 mg/kg IV every 2 weeks;**
Leucovorin 400 mg/m2 iv over 2 hrs before 5-FU d1
5-FU 400 mg/m2 iv bolus d1 followed by 2400 mg/m2 iv over 46 hrs
Oxaliplatin (Eloxatin) 85 mg/m2 iv d1
Q2w x 12 cycles

**Bev + XELOX**

**Bevacizumab 5 mg/kg IV every 2 weeks;**
Capecitabine (Xeloda) 1000 mg/m2 po bid x 14 days
Oxaliplatin (Eloxatin) 130 mg/m2 iv over 2 hrs d1
Q3w x 8 cycles

**Appendix II. Multivariable Model of Factors Associated with Receiving >70% RDI^a^**

|  | **OR** (95% CI) | **P value** |
| --- | --- | --- |
| **Chemotherapy Regimens** |  |  |
| 5-FU/ LV | 1.00 |  |
| Oxaliplatin plus 5-FU/LV | 1.63 (0.85,3.13) | 0.14 |
| Oxaliplatin plus capecitabine | 0.36 (0.12,1.09) | 0.07 |
| Capecitabine monotherapy | 0.27 (0.12,0.61) | 0.002 |
| Mixed/ Other | 0.49 (0.22,1.11) | 0.09 |
| **Age** (years) |  |  |
| <55 | 0.34 (0.14, 0.85) | 0.02 |
| 55-64 | reference |  |
| 65-74 | 0.46 (0.24, 0.90) | 0.02 |
| 75+ | 0.31 (0.15, 0.65) | 0.002 |
| **Male** | 0.29 (0.03,3.13) | 0.31 |
| **Race/Ethnicity** |  |  |
| White (non-Hispanic) | 1.00 |  |
| Hispanic | 1.63 (0.69,3.85) | 0.26 |
| Black (non-Hispanic) | 0.87 (0.42,1.79) | 0.70 |
| Other/missing | 1.64 (0.37,7.24) | 0.52 |
| **Charlson Comorbidity Index** | 1.07 (0.91,1.26) | 0.42 |
| **Married** | 0.66 (0.39,1.12) | 0.12 |
| **Node, N2 vs. N1** | 0.77 (0.37,1.62) | 0.50 |
| **Number of Positive Lymph Nodes** | 0.98 (0.90,1.07) | 0.64 |
| **Lymphovascular Invasion** |  |  |
| No | 1.00 |  |
| Yes | 1.19 (0.62,2.29) | 0.60 |
| Unknown/ missing | 1.58 (0.83,3.00) | 0.16 |
| **ECOG Performance Status** |  |  |
| 0 | 1.00 |  |
| 1 | 0.89 (0.35,2.26) | 0.81 |
| 2 | 0.51 (0.15,1.73) | 0.28 |
| Missing or unknown | 0.53 (0.26,1.06) | 0.07 |

5-FU=fluorouracil; ECOG=Eastern Cooperative Oncology Group

^a^The multivariable model includes predictor variables that were suggestive of a bivariate association (i.e., P<0.15). Male sex, married and Charlson Comorbitiy Index were forced into the model.

**Appendix III. Number of Adverse Drug Events and Rate per 10 Cycles by Regimen^a^**

| **Adverse Drug Event** | **Total^†^**  N=367  patients  (2653 cycles) | | | **5-FU/ LV**  N=158  patients  (591 cycles) | | **Oxaliplatin**  **plus**  **5-FU/LV**  N=169  patients  (1453 cycles) | | **Oxaliplatin plus capecitabine**  N=40  patients  (1844 cycles) | | **Capecitabine monotherapy**  N=58  patients  (312 cycles) | | **Other**  N=26  patients  (113 cycles) | |
| --- | --- | --- | --- | --- | --- | --- | --- | --- | --- | --- | --- | --- | --- |
|  |  |  |  |  |  |  |  |  |  |  |  |  |  |
|  |  |  |  |  |  |  |  |  |  |  |  |  |  |
|  | # of  patients | # of ADEs | Rate  per 10 Cycles | # of ADEs | Rate per 10 Cycles | # of ADEs | Rate  per 10 Cycles | # of ADEs | Rate  per 10 Cycles | # of ADEs | Rate  per 10 Cycles | # of ADEs | Rate  per 10 Cycles |
| **Neutropenia**^b^ | 86 | 154 | 0.58 | 29 | 0.49 | 109 | 0.75 | 6 | 0.33 | 1 | 0.03 | 8 | 0.71 |
| **Diarrhea/gastrointestinal toxicity** | 100 | 134 | 0.51 | 49 | 0.83 | 37 | 0.25 | 13 | 0.71 | 21 | 0.67 | 14 | 1.24 |
| **Thrombocytopenia** | 59 | 114 | 0.43 | 8 | 0.14 | 98 | 0.67 | 6 | 0.33 | 0 | 0 | 2 | 0.18 |
| **Neuropathy** | 44 | 52 | 0.2 | 5 | 0.08 | 40 | 0.28 | 4 | 0.22 | 1 | 0.03 | 2 | 0.18 |
| **Hand-foot syndrome** | 23 | 29 | 0.11 | 3 | 0.05 | 2 | 0.01 | 1 | 0.05 | 23 | 0.74 | 0 | 0 |
| **Stomatitis/mucositis** | 26 | 28 | 0.11 | 15 | 0.25 | 6 | 0.04 | 0 | 0 | 4 | 0.13 | 3 | 0.27 |
| **Clostridium difficile infection** | 19 | 25 | 0.09 | 9 | 0.15 | 9 | 0.06 | 2 | 0.11 | 2 | 0.06 | 3 | 0.27 |
| **Fatigue/weakness** | 12 | 14 | 0.05 | 3 | 0.05 | 7 | 0.05 | 1 | 0.05 | 2 | 0.06 | 1 | 0.09 |
| **Rash** | 10 | 13 | 0.05 | 1 | 0.02 | 5 | 0.03 | 1 | 0.05 | 4 | 0.13 | 2 | 0.18 |
| **Acute renal dysfunction** | 9 | 12 | 0.05 | 3 | 0.05 | 4 | 0.03 | 1 | 0.05 | 1 | 0.03 | 3 | 0.27 |
| **Decline in performance status** | 8 | 10 | 0.04 | 2 | 0.03 | 6 | 0.04 | 1 | 0.05 | 1 | 0.03 | 0 | 0 |
| **Elevated liver function tests** | 8 | 8 | 0.03 | 3 | 0.05 | 0 | 0 | 2 | 0.11 | 3 | 0.1 | 0 | 0 |
| **Thromboembolism** | 7 | 8 | 0.03 | 1 | 0.02 | 6 | 0.04 | 0 | 0 | 1 | 0.03 | 0 | 0 |
| **Anemia** | 5 | 7 | 0.03 | 1 | 0.02 | 3 | 0.02 | 1 | 0.05 | 0 | 0 | 2 | 0.18 |
| **Small bowel obstruction** | 4 | 4 | 0.02 | 3 | 0.05 | 0 | 0 | 1 | 0.05 | 0 | 0 | 0 | 0 |
| **Cardiac vasospasm** | 1 | 2 | 0.01 | 0 | 0 | 2 | 0.01 | 0 | 0 | 0 | 0 | 0 | 0 |
| **Other** | 37 | 42 | 0.16 | 9 | 0.15 | 23 | 0.16 | 1 | 0.05 | 5 | 0.16 | 4 | 0.35 |
| **Unknown** | 4 | 4 | 0.02 | 0 | 0 | 3 | 0.02 | 1 | 0.05 | 0 | 0 | 0 | 0 |
| **Total** | 259 | 660 | 2.49 | 144 | 2.44 | 360 | 2.48 | 42 | 2.28 | 69 | 2.21 | 44 | 3.89 |

^a^A patient could have more than 1 ADE. ADEs were linked to the regimen that the patients actually were on at the time. The numbers of patients (N) and cycles were calculated accordingly for each regimen.

^b^1 ADE has an unknown regimen
